# Arthropod Communities in Urban Agricultural Production Systems under Different Irrigation Sources in the Northern Region of Ghana

**DOI:** 10.3390/insects11080488

**Published:** 2020-08-01

**Authors:** Louis Amprako, Kathrin Stenchly, Martin Wiehle, George Nyarko, Andreas Buerkert

**Affiliations:** 1 Organic Plant Production and Agroecosystems Research in the Tropics and Subtropics (OPATS), University of Kassel, Steinstrasse 19, D-37213 Witzenhausen, Germany; amprako@outlook.com (L.A.); stenchly@uni-kassel.de (K.S.); buerkert@uni-kassel.de (A.B.); 2 Competence Centre for Climate Change Mitigation and Adaptation (CliMA), University of Kassel, Kurt-Schumacher-Straße 25, D-34117 Kassel, Germany; 3 Grassland Science and Renewable Plant Resources (GNR), University of Kassel, Steinstrasse 19, D-37213 Witzenhausen, Germany; 4 Tropenzentrum-Centre for International Rural Development, University of Kassel, Steinstrasse 19, D-37213 Witzenhausen, Germany; 5 International Center for Development and Decent Work, University of Kassel, Kleine Rosenstrasse 1-3, D-34109 Kassel, Germany; 6 Department of Horticulture, Faculty of Agriculture, University for Development Studies (UDS), P.O. Box TL 1882, Tamale, Ghana; gnyarko@uds.edu.gh

**Keywords:** agrobiodiversity, ecosystem service, feeding guild, insects, NMDS, RDA, species function, Sub-Sahara Africa, urban agriculture

## Abstract

Urban and peri-urban agricultural (UPA) production systems in West African countries do not only mitigate food and financial insecurity, they may also foster biodiversity of arthropods and partly compensate for structural losses of natural environments. However, management practices in UPA systems like irrigation may also contribute to disturbances in arthropod ecology. To fill knowledge gaps in the relationships between UPA management and arthropod populations, we compared arthropods species across different irrigation sources in Tamale. During a 72-h sampling period, 14,226 arthropods were caught with pitfall traps and pan traps from 36 fields. These specimens comprised 13 orders, 103 families, 264 genera, and 329 taxa (243 identified species, 86 unidentified species) and categorized into five feeding guilds (carnivores, decomposers, herbivores, omnivores, and pollinators). Species richness, species accumulation curves, and diversity functions (richness, evenness, and dispersion) were calculated to characterize the arthropod community. Non-metric multidimensional scaling was applied to examine structural similarity of arthropod communities among sites. To account for the effects of soil-related data, we furthermore applied a redundancy analysis. Arthropods grouped according to the irrigation water source, whereby the dipterans were most dominant under wastewater conditions. Here, particularly the eye gnat, *Hippelates pusio*, a disease-causing vector for humans, accounted for the dipterans. The occurrence of three alien ant species suggested community shifts through invasive species, while the occurrence of seven ant species (at least one ant species occurred under each water source) that form mutualistic relationships with aphids highlighted future risks of aphid pest outbreak. Future studies on these taxa should specifically target their ecological and economic effects and potential countermeasures.

## 1. Introduction

Agriculture practiced within and around human agglomerations known as urban and peri-urban agriculture (UPA) has been a source of food for humans for centuries [1]. Its role during the last two decades has grown due to its contribution to food security and the improvement of livelihoods.

Food and financial security have been a global concern for many decades, especially in the developing world where food insecurity risk is high, and cascading effects like mass migration and social conflicts within affected areas prevail. Africa is a region where one-fifth of the population experiences hunger [2], and in Sub-Saharan Africa, almost half of the people live below the poverty line [3]. Under these conditions, UPA helps to mitigate hunger and poverty in the urban population and thus contributes to the resilience of African cities [4].

Since UPA systems are productive vegetative features, they may also increase the arthropod biodiversity of urban landscapes and improve ecosystem services, ameliorate urban-heat-island, and serve as habitats for wildlife [5]. Moreover, these typically small-scale land-use systems not only support urban biodiversity, their productivity, in turn, also heavily depends on the biodiversity of diverse pollinating insects and pest regulating agents [6]. It is in this context that ecologists are interested in collecting field data to better understand the diversity of arthropod communities and how these are determined by agricultural intensification and other environmental conditions.

Agricultural intensification has been shown to influence the species composition of communities and negatively affect their functional trait diversity and thus contributes to the attenuation of ecosystem functioning and ecosystem services [7]. In and around West African cities, agricultural intensification leads to nutrient applications far beyond the optimum [8]. High fertilization rates are often combined with high doses of pesticides. Additionally, because of the long lean season, West African UPA farmers mostly rely on external water sources other than rainfall to irrigate their crops, whereby some farmers resort to the usage of untreated municipal wastewater (WW) from open channels. Such water sources often run through cities and carry a mixture of natural dilutes and human waste, laden with chemicals and pathogens. Irrigation water may also be a medium for the pupation of insect pests [9] and disease vectors, such as mosquitoes of the genus *Anopheles* and *Culex* [10]. As such, the usage of inadequately processed wastewater for irrigation that, in turn, can pose major threats to soil properties [11], as well as below and above-ground biodiversity [12], may negatively affect farmers’ and consumers’ livelihoods in West Africa. In addition, the shift in agricultural land use from wind-pollinated crops, such as sorghum (*Sorghum bicolor* Moench), millet (*Pennisetum glaucum* (L.) R.Br.), and maize (*Zea mays* L.), to crops that rely on insect pollination for seed and fruit development, such as okra (*Abelmoschus esculentus* (L.) Moench), pepper (*Capsicum annum* L.), and garden eggs (*Solanum aethiopicum* L.) [13], has led to increased dependence of West African farmers’ livelihoods and food security on the existing biodiversity [14]. Cities are hotspots of biological invasions. Particularly semi-natural urban ecosystems, such as UPA systems, can assist in the invasion of non-native species, which negatively affect ecosystem services upon which human societies depend [15]. They may further affect human health by acting as vectors of human and animal diseases, causing allergic reactions [16], and displace native species, thereby contributing to the homogenization or even to the loss of species within cities [17,18].

Despite these contributions, little is known about species composition of arthropod communities and their ecological role in West African urban agroecosystems and how these arthropod communities are affected by farmers’ management practices, such as irrigation sources.

This study, therefore, aimed at conducting a baseline species inventory of arthropods inhabiting West African urban production systems and assessing how arthropod communities are shaped by farmers’ practices, considering the use of different sources of irrigation water.

## 2. Materials and Methods

### 2.1. Study Area

The Northern Region of Ghana, with its capital Tamale, is a rapidly developing province. It has a tradition of UPA with a positive impact on household food security as it provides access to food and generates income. In Tamale, UPA activities are the third most important source of earnings, after informal business and wages, and contribute nearly a fifth to a household’s income [19]. UPA in and around Tamale takes many forms, with various crop farm types characterized by different spatial and tenure arrangements, and different sources of irrigation [4]. Irrigation water is a limiting factor in this Sudanian region. Notwithstanding, Tamale has the least available groundwater in the entire country due to its highly weathered soils that have a high water permeability and reduce the storage capacity of aquifers [20]. The region is the poorest in Ghana [21], and tap water is available to just about 50% of the urban dwellers [22]. In this context, UPA farmers resort to rainwater, tap water, well water, dam water, and municipal wastewater to irrigate crops depending on its proximity or availability. The UPA systems constitute a mosaic of small fields in which vegetables like cabbage (*Brassica* spp. L.), lettuce (*Lactuca sativa* L.), amaranth (*Amaranthus* spp. L.), okra (*Abelmoschus esculentus* (L.) Moench), sweet pepper (*Capsicum annum* L.), spring onion (*Allium* spp. L.), garden eggs (*Solanum aethiopicum* L.), tomatoes (*Solanum lycopersicum* L.), carrots (*Daucus carota* (Hoffm.) Schübl. & G. Martens), and jute mallow (*Corchorus* spp. L.) are grown in polycropping systems.

**Figure 1:**
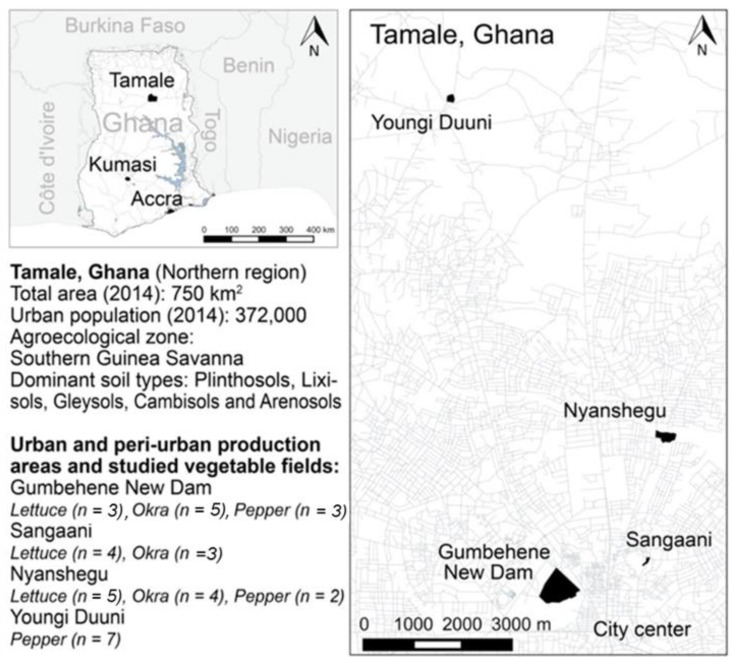
Map of Ghana with major cities (**left**) and Tamale in the Northern Region of Ghana (**right**) showing the location of the studied urban and peri-urban production systems. Data retrieved from www.openstreetmap.org (OpenStreetMap contributors 2019). Figure produced in R version 3.6.0 (R Development Core Team 2018). Field-level coordinates are available as a supplementary kmz file.

This study was conducted in and around Tamale. The local climate is classified as semi-arid with annual mean minimum and maximum temperatures of 26 °C and 31 °C, respectively, and a unimodally distributed monthly precipitation between 2 and 231 mm (sum: 1111 mm) per year peaking between August and October (1982–2012, https://de.climate-data.org). Arthropod data were collected in the suburbs (locations) of Nyanshegu (9°25′22.8″ N, 0°50′6″ W), Gumbehene New Dam (9°14′52.8″ N, 0°10′19.2″ W), Sangaani (9°25′8.4″ N, 0°50′20.4″ W), and Youngi Duuni (9°14′52.8″ N, 0°42′43.2″ W). Between seven and eight vegetable (pepper, okra, and lettuce) fields were distributed across locations, comprising a total of 36 vegetable fields (Figure 1), with an average size of 300 m^2^.

### 2.2. Site Description

Across the 36 fields, a set of parameters related to farmer’s management recorded such types of amendments and irrigation water, farm age, and pesticide and herbicide usage. Besides, the topsoils’ (0–20 cm) electrical conductivity (EC), carbon-to-nitrogen ratio (C/N), total phosphorous (P), and pH were also determined. To further characterize location-specific differences, the most probable number (MPN) of fecal indicator bacteria—*Escherichia coli* and *Enterococcus* spp.—per 100 mL of each irrigation source was sampled once in August 2016 and analyzed using standard protocols. Additionally, a 200 m long transect (north-south direction) was established in the center of each of the four locations to count all perennial species with >5 cm diameter at breast height 10 m left and right each of the transects and identified to the species level. Perennial species richness and diversity indices (Exponential Shannon index, Inverse Simpson’s index, Inverse Berger–Parker index) based on Hill numbers [23] were calculated with the ‘hillR’ and ‘diverse‘ packages [24,25] in R version 3.6.2 “Dark and Stormy Night” (R Development Core Team, 2019).

The sources of irrigation water differed markedly per location (Table 1). In *Youngi Duuni,* the least urbanized location, agriculture was purely rainfed, while crop cultivation in *Gumbehene New Dam,* a farming area located on the periphery of a protected forest, relied on tap water irrigation. Irrigation with a tap was possible due to the proximity of this farming location (less than 200 m away) to the Ghana Water Company Limited, which provides water to the region. The *Sangaani* fields were irrigated with water from a well, while the farming location of *Nyanshegu* relied on water from an open (wastewater) channel.

Most of the fields were amended with mineral fertilizers: 100% of rainfed fields, about 80% of wastewater and tap-irrigated fields each, and half of the well water irrigated fields. In contrast, manure fertilization was uncommon (28% of fields) and applied by 50% of the well water fields, by 20% of the rainfed and wastewater fields each, and by 10% of the tap irrigated fields. All types of irrigation water were contaminated with the fecal indicator bacteria *Escherichia coli* and *Enterococcus* spp., with the highest loads in wastewater. Wastewater usage significantly increased soil C/N ratio, EC, Bray-P (Table 1).

For the four sampled UPA locations, tree abundance ranged between 73 and 125 individuals (Table S1). The rainfed location of *Youngi Duuni*, approximately 15 km from the city center (linear distance), had the highest tree species richness, whereas richness was lowest for tap irrigated fields of *Gumbehene New Dam*, located next to a forest reserve in Tamale’s city center (Table 2). The well water irrigated fields of *Sangaani* and wastewater irrigated fields of *Nyanshegu* instead exhibited 11 and 13 species, respectively. At all four locations, mango (*Magnifera indica* L.), neem (*Azadirachta indica* A.Juss), and lebbeck (*Albizia lebbeck* (L.) Benth) were the most common tree species (Table S1). The tree Exponential Shannon index among locations was more balanced (5.2–6.9) than the Inverse Simpson’s and the Inverse Berger–Parker index (3.3–5.7 and 1.9–3.3, respectively) (Table 2).

### 2.3. Arthropod Collection and Species Functions

For arthropod sampling, two types of traps (pitfall and pan trap) were used in August 2016 (rainy season). Ground active arthropods were sampled with a set of five roofed pitfall traps per plot on a 5 × 5 m grid (one trap in the center; diameter top opening = 12 cm, depth = 11 cm; *n =* 180), while the flying arthropods were sampled using a line of four pan traps (*n =* 144) along a 5 m transect within a field. Each pan trap consisted of three plastic soup bowls attached to a stake at a one-meter height above ground. Each bowl had a depth of 3 cm and was sprayed with white, yellow, and blue UV-reflecting colors (Sparvar Leuchtfarbe, Spray-Color GmbH, Merzenich, Germany), respectively, to catch Diptera, Hymenoptera, and Hemiptera, which are particularly attracted by the UV spectrum. Salt-saturated water with a drop of detergent soap (Dishwashing Liquid Sensitive, Sonett GmbH, Deggenhausen, Germany) was poured into the trap liquid. Pitfall and pan traps were exposed for 72 h each, after which all arthropods were deposited in 70% alcohol and determined to the species level. Arthropod species sampled were determined with arthropod taxa-specific literature and later cross-checked against the GBIF database [26] for recent taxonomic changes. All verified entries were classified into one of five feeding guilds, namely, carnivores, decomposers, herbivores, omnivores, or pollinators, based on intensive online literature and database searches. Carnivores comprised arthropods that relied primarily on other arthropods for food during their adult stage (or during their larval stage if they do not feed as adults). Decomposers comprised arthropods that obtained their diet from decaying organic matter, while herbivores obtained their diet by feeding on any part of a plant (including seeds). Omnivores referred to those arthropods that fed on other animal species, organic matter, and plants, while pollinators were those that fed on pollen and or nectar and pollinated flowers as a result.

### 2.4. Species Indices and Multivariate Statistics

To evaluate the reliability of the sampling procedure, rarefaction curves by means of the Mao Tao estimator [27] were generated with EstimateS [28]. All further calculations and visualizations were done with R version 3.6.2 “Dark and Stormy Night” (R Development Core Team, 2019). The overall share of arthropod orders was displayed using the ‘treemap’ package [29], and arthropod abundance data were analyzed with the ‘vegan’ package [30]. As for tree abundance, arthropod richness and diversity indices (Exponential Shannon index, Inverse Simpson’s index, Inverse Berger–Parker index) based on Hill numbers [23] were calculated with the ‘hillR’ package. To describe arthropod communities’ function, evenness, and dispersion, the ‘functional diversity’ (FD) package [31] was used in R by linking a species-abundance matrix with a species-function (feeding guild) matrix, giving equal weight to all functions. The community-weighted mean of a trait (CWM hereafter), defined as the field-level trait presence weighted by species abundances, was calculated to characterize arthropod communities [32]. Since data were not normally distributed, Kruskal–Wallis following by post hoc Conover–Iman tests (when necessary) were performed to assess the difference between the mean FD indices and CWMs for each irrigation source.

Thereafter, all arthropods with a frequency of at least 0.1% and an occurrence in at least three of the four types of irrigation were selected, resulting in a total of 24 species (64% of the total abundance of species studied). The analyses of only a fraction of sampled species are not untypical in ecological studies as it reduces noise and allows the drawing of more meaningful relationships [32,33]. Boeckel and Baumann [34], for instance, selected only the most abundant species in their study of phytoplankton in the South Atlantic Ocean. Morales and Aizen [35] and Gamito [36] reduced species communities when analyzing plant-pollinator interactions and benthic communities, respectively, to improve the ordination display. To reduce the effect of largely differing abundances of the 24 species involved, abundance data were Hellinger-transformed. Afterward, the ‘arthropod species—irrigation source’ matrix was subjected to non-metric dimensional scaling (NMDS) of unconstrained ordination [37]. The stress (Kruskal’s goodness-of-fit index) value was used to evaluate the goodness of the NMDS. A non-parametric permutational multivariate analysis of variance (bootstrapped perMANOVA test) based on 999 permutations accompanied by the Levene’s variance homogeneity test was performed to compare the arthropods sampled under the different irrigation sources. The arthropod species-irrigation matrix (response data) was also subjected to a redundancy analysis (RDA), using the soil parameters, C/N, EC, P, and pH, as explanatory variables. The amount of variance explained by the explanatory variables was evaluated based on adjusted R^2^ statistics.

## 3. Results

### 3.1. Arthropod Communities’ Structure

**Figure 2:**
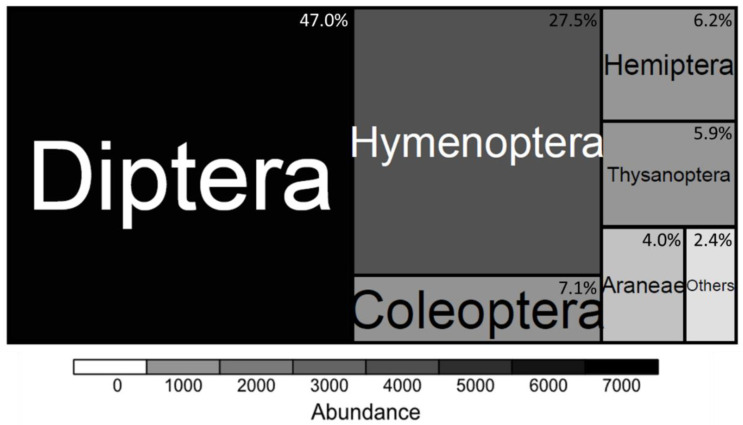
The abundance of arthropod orders caught with pitfall and pan traps during a 72-h sampling in 36 vegetable fields of Tamale, Northern Region, Ghana, in August 2016.

A total of 14,226 arthropod individuals (13 orders, 103 families, 264 genera, and 329 taxa) were sampled, from which 12,089 (85% of the total arthropods) comprising 243 species (12 orders, 88 families, 205 genera) were successfully determined, while 86 taxa (15% of the total arthropods, 10 orders, 55 families) could only be determined to the genus level (Table S2). The most abundant orders were the Diptera, the Hymenoptera, and the Coleoptera (Figure 2). Individuals belonging to the orders Dermaptera, Dictyoptera, Glomerida, Isopoda, Lepidoptera, and Orthoptera accounted for about 2.4% of the sampled arthropod community and were grouped as ‘Others’ (Figure 2). The most abundant families were the Chloropidae (Diptera; *n =* 6123, 43%), Formicidae (Hymenoptera; *n =* 3698, 26%), Thripidae (Thysanoptera; *n =* 674, 5%).

The most abundant species was the eye gnat (*Hippelates pusio*, Chloropidae, Diptera; *n =* 5011, 35% of all arthropods). Apart from the arthropods, two Haplotaxida species were caught (*Eisenia fetida* SAVIGNY, 1826, *n =* 4, 0.03%; *Eudrilus eugeniae* KINBERG, 1876, *n =* 3, 0.02%).

**Figure 3:**
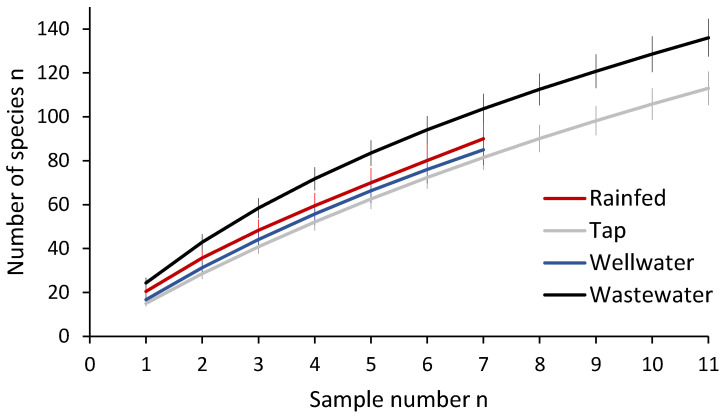
Arthropod species accumulation curves (Mao Tao estimator ± standard deviation) of vegetable fields under different irrigation sources (four urban and peri-urban agriculture (UPA) locations) of Tamale, Northern Region, Ghana in August 2016.

Overall, arthropod species accumulation curves for the different irrigation water sources differed significantly for the tap and the wastewater sources, but none of the curves plateaued (Figure 3).

The wastewater irrigated fields captured the highest abundance and richness, while the rainfed and well water irrigated fields had the highest diversity indices (Table 3). Functional richness and diversity (evenness and dispersion) in all four locations were low and rather homogenously distributed (Table 4). Although statistically also non-significant, CWM of the different guilds indicated that carnivores and decomposers tended to be absent, while omnivores were present under rainfed conditions (Table 4). Herbivores and pollinators were unaffected by irrigation type.

### 3.2. Effect of Farmers’ Management on Arthropod Species

Out of the shortlisted 24 taxa, a strong association (higher than 60% occurrence per taxa) with wastewater was found for *Agelenopsis aperta*, *Ootheca mutabilis*, and *Tetrix subulata* (Table 5). Dominant in well water irrigated fields were *Calliphora* sp., *Aspavia armigera*, and *Chironomus plumosus*, while for tap water, *Hermetia illucens*, *Sarcophaga carnaria*, *Camponotus* sp., and *Megachile latimanus* prevailed. *Zophopetes dysmephila* dominated rainfed fields.

**Figure 4:**
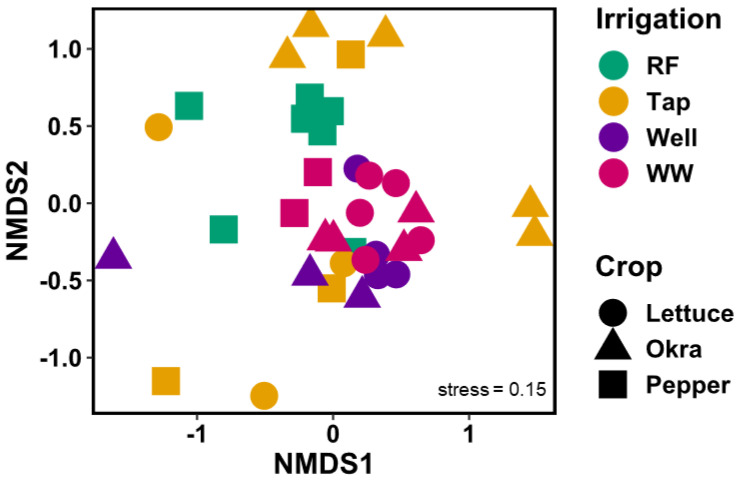
Non-metric multidimensional scaling (NMDS) biplot based on Bray–Curtis dissimilarity of all 36 arthropod communities caught on vegetable fields considering irrigation source, namely, rainfed (RF; green), tap water (Tap; yellow), well water (Well; purple), wastewater (WW; magenta) and crop type by using the 24 most abundant and equally distributed species sampled in UPA systems in Tamale, Northern Region, Ghana in August 2016.

The NMDS biplot (stress = 0.15, k = 3; Figure 4) shows that irrigation affected arthropod species more than crop type; however, both parameters were statistically significant (*p* = 0.001 and *p* = 0.029, respectively). Particularly rainfed and wastewater clustered with almost no overlap (*p* = 0.003 perMANOVA). The test of homogeneity of variance was equal for crops (*p* = 0.182, homogeneity given) but unequal for water sources (*p* = 0.001, homogeneity not given). Other assessed management-related parameters had no influence on the arthropod community.

**Figure 5:**
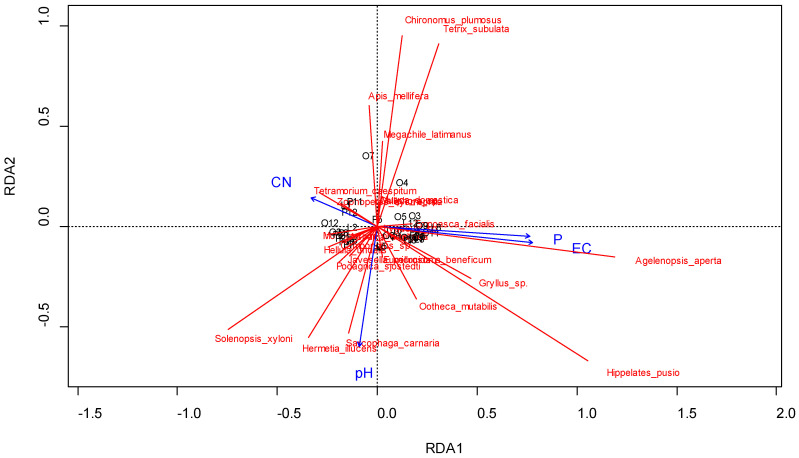
RDA (redundancy analysis) triplot (scaling 1) showing the relationship between soil carbon-to-nitrogen ratio (C/N), soil electric conductivity (EC), soil phosphorous content (P), and soil pH to the shortlisted 24 arthropod species sampled for 72 h in UPA systems of Tamale, Northern Region, Ghana, in August 2016.

Some of the 24 shortlisted species were dependent on the soil-related parameters as visualized by the RDA (Figure 5). The occurrence of *Agelenopsis aperta* reflected an increase in P and EC, while the number of *Hermeticia illucens*, *Sarcophagia carnaria*, and *Solenopsis xyloni* correlated with an increase in pH. *Gryllus* sp., *Hippelates pusio*, and *Ootheca mutabilis* seemed to be intermediately dependent on all three soil parameters, but were in general related to higher nutrient loads (opposed to C/N, which indicates an excess of nitrogen). The presence of *Tetramorium caespitum* and *Zophopetes dysmephilia* moderately corresponded to an increase of C/N. Species like *Apis mellifera*, *Chironomus plumosus*, *Megachile latiamanus*, and *Tetrix subulata* appeared to be less affected by soil C/N, EC, and P and responded to rather low pH values. However, the model (R^2^ adjusted) explained only 2.3% of the variation and was non-significant (*p* = 0.25).

## 4. Discussions

### 4.1. Richness and Function of Arthropod Species in the Context of UPA-Systems

Studies on arthropods’ functional role within UPA systems, as, for instance, their contribution to improving and stabilizing crop-pollination services, are still rare [38]. Particularly in West Africa, where UPA systems play a key role in improving the nutritional security of vulnerable populations [39], knowledge of crops-associated species communities and their functional (beneficial) role is limited. Given the growing economic importance of West African UPA activities, which comprise the cultivation of more and more crops that require animal-mediated services, such as pollination and predation [40], a better understanding of the effects of land-use intensity and irrigation water quality on biodiversity-based ecosystem services is needed. Filling this knowledge gap is urgent as West African systems are increasingly exploited by the excessive and uncontrolled application of potentially harmful pesticides. Thus, studies on the functional community ecology of crop-associated insects should comprise a more comprehensive biodiversity monitoring (pollinator efficiency, predators’ prey spectrum) while ensuring that different life stages of all taxa are taken into consideration [41]. This knowledge may allow the implementation of more sustainable management practices in UPA systems.

Among the few available studies on arthropods in Northern Ghana, Agyen-Sampong [42] described 25 families and 46 genera of arthropod pests of sorghum in Nyankpala. N’Djolossè et al. [43] recorded 8 orders, 36 families, and 56 genera of arthropods associated with shea trees (*Vitellaria paradoxa* C.F.Gaertn.) in Northern Ghana, while a study in the Kogyae Strict Nature Reserve (Forest Savanna Transition zone) recorded 21 orders, 135 families, including 107 butterfly species [44]. These values are in agreement with our data even if one would have expected that overall species richness would be lower in our region than in the more humid zones studied in the aforementioned regions. The obtained species accumulation curves suggest that even more species could be caught with more intensive sampling.

### 4.2. Effects of Irrigation Sources on Arthropod Community Structure

Almost a quarter of the UPA farmers in the Northern Region strongly relies on the usage of municipal untreated wastewater as the only source for irrigation, particularly for lettuce and okra production. From a farmer’s perspective, the use of municipal wastewater in UPA is highly advantageous, as its content of inorganic and organic dissolved substances makes wastewater a free fertilizer source [45]. Depending on its quality and origin, regular wastewater irrigation may restrain plant growth [46] and also change soil properties [47,48]. Furthermore, the usage of wastewater in agricultural production can directly and indirectly affect arthropod communities, below and aboveground. Aboveground effects can be related to changing plant chemical compositions, irregular flower formation, or changed nectar composition repelling pollinating insects [49]. In our study, soils that received high doses of wastewater had a significantly higher EC, C/N ratio, and P-Bray content and the highest soil pH (Table 1), therefore, likely influencing belowground arthropod communities.

Arthropod abundance was highest in wastewater irrigated fields and lowest under rainfed and well water conditions. A possible explanation for this may be the higher quantity and quality of the foliage, stimulated by nutrient-rich wastewater, thereby attracting herbivorous insects, as observed in Ouagadougou, Burkina Faso by Stenchly et al. [13], who recorded high abundances of orthopterans and hymenopterans in spinach fields (*Spinacia oleracea* L.) irrigated with wastewater. Another explanation could be the well-developed olfactory system of insects [50], which allows the rapid detection of carbohydrates in irrigation wastewater [51]. A further explanation may be related to the low tree abundance recorded, which is known to decrease microhabitat conditions for arthropod predators, such as bats and birds, but also arthropods, such as spiders, all which potentially regulate arthropod communities [52]. In spite of the high arthropod abundance and richness in wastewater irrigated fields, arthropod communities under these conditions are dominated by a few species only, as revealed by the low values of the Exponential Shannon, the Inverse Simpson’s, and the Inverse Berger–Parker indices. Silva et al. [53] found that amending fields with mineral fertilizers increased the abundance of arthropod predators, while other feeding guilds declined. Despite the plausibility of this explanation, none of our results on the functional richness and CWM indicated that this was valid for our study. Instead, the guild of decomposers had the lowest CWM under rainfed and the highest one under well water conditions. The decomposer species *Chironomus plumosus* (Dipterans, Chironomidae) was found especially under well- and wastewater conditions. Chironomidae are known to prefer decaying organic amendments [54]. Considering only the CWM values for decomposers may indicate the usefulness of this guild as an indicator group for non-contaminated soil and water conditions and, therefore, ecosystem health in UPA systems. Bundschuh et al. [55] showed a reduced invertebrate decomposer activity in the presence of chemicals in wastewater irrigation.

### 4.3. Ecology of the Dominant Arthropod Species

The alien eye gnat (*Hippelates pusio*, Diptera), which is a vector for bovine mastitis [56], anaplasmosis [57], bacterial conjunctivitis [58], and yaws [59], dominated our samples to almost 50%. To our knowledge, this is the first time a study highlights the association between wastewater irrigation and the occurrence of eye gnats in Tamale, and further studies should investigate how this may be related to the incidence of this vector transmitted diseases. From our data, *Hippelates pusio* was strongly correlated with the use of wastewater, amounting to 58% occurrence in wastewater irrigated fields of Nyanshegu (Table 5). Yaws is a skin and bone disease caused by the spirochete bacterium *Treponema pallidum pertenu* Schaudinn & Hoffman. The disease was controlled in the late 1960s in Ghana, but it resurged in the early 1980s [60]. It is still prevalent in Ghana and is even believed to have developed resistance to antibacterial drugs [61]. Anaplasmosis, a disease caused by the bacteria genus *Anaplasma*, causes leukopenia, thrombocytopenia, and anemia in livestock and fevers in humans [57]. It was found to have an incidence of 60% in livestock in Ghana’s capital Accra [62]. Given that livestock production is more common in the north of Ghana [63], it is plausible that the anaplasmosis incidence would be higher also in our study region than in the more developed southern capital area.

While the eye gnat dominated in fields with problematic hygiene, two out of three alien ant species (Formicidae, Hymenoptera) dominated in rainfed fields. Among those was the most abundant ant species—the fire ant (*Solenopsis xyloni*)—belonging to the *Solenopsis geminate* species group [64], and a native to the USA. Due to the species’ highly carnivorous feeding behavior that may cause destabilization in community patterns of important predatory arthropods on agricultural fields, this species has a great potential to become a serious pest [65]. As such, it poses a great risk of becoming invasive in West African UPA systems. Furthermore, *Solenopsis xyloni* attends not only to aphids and leafhoppers for their honeydew [66] but also causes considerable damage to seed banks, attacks newly hatched birds, girdles agricultural plants, and affects agricultural produce [67]. The origin of the second most dominant ant—the Pharaoh ant (*Monomorium pharaonis*)—is uncertain and was ascribed to South East Asia [65] as well as to tropical Africa. This species also dominated the rainfed fields (53%), and although its influence on agriculture is yet to be determined, its abundance is associated with urbanization [68], and it is believed to spread diseases in hospitals [69]. Although only 12% of the Pharaoh ants were sampled in the tap irrigated fields of *Gumbehene New Dam*, which are among the most urbanized areas of Tamale, the species’ potential to cause problems is high as the third largest teaching hospital of Ghana is just about four kilometers from these fields. The third most dominant species—the sugar ant (*Tetramoroum caespitum*)—is native to Europe [70]. It is known to avoid urban areas; however, the exact reason for this non-synanthrope behavior is unknown [71]. *Tetramoroum caespitum* that dominated wastewater irrigated fields (58%) may thus still be of concern for farmers in less urbanized suburbs of Tamale since it is also known to protect aphids from biological predators [72]. All three highly aggressive ant species of exotic origin, which were found to dominate arthropods’ composition within UPA fields, may have negative effects on native arthropod communities and their functions, and they become invasive in the future. The three alien ant species accounted alone for nearly 16% of the sampled arthropod community.

Apart from the dominance of some invasive species, one pest regulatory species, the desert spider *Agelenopsis aperta*, occurred in 87% of the wastewater irrigated fields. A plausible explanation could be the lush growth of crops through nutrient-rich wastewaters, which attracted abundant prey for this species. Pommeresche et al. [73] and Utami et al. [74] reported a high correlation between soil amendments with the abundance and diversity of soil fauna, while Akamatsu et al. [75] found that insects attracted to wastewater discharge are potential feed sources for spiders.

The occurrence of ants and lepidopterans are also of agricultural concern. At least seven ant species were found within the studied UPA systems (*Camponotus atriceps* and *C. chrysurus*—only in tap water irrigated fields*, C. importunes*—dominated in rainfed fields (73%), *Monomorium biocolor* –only in well irrigated fields, *M. pharaonis*—dominated in rainfed (53%) and well irrigated fields (34%), *Odontomachus brunneus*—dominated in well water irrigated fields (95%), and *Tetramorium caespitum*—dominated in well water irrigated (58%) and rainfed fields (24%)), which form mutualistic relationships with aphids. Aphid-ant mutualism has serious consequences for food security as they reduce crop yield while depleting pest regulator populations by up to 50% [76]. This mutualistic relationship is further strengthened by urbanization [77], as prevalent in the Northern Region of Ghana. Lepidopterans like *Colotis* sp., *Dyptergia* sp. and coleopterans like *Podagrica sjostedti* and *P. uniformis* are herbivores that may be of further concern to farmers, although their sampled abundances were low in this study.

### 4.4. Evaluation and Reliability of the Data

The qualitative assignment of arthropods to feeding guilds is methodologically problematic. Lepidopterans have different feeding strategies and intensities at different life stages. While the juvenile larva feeds on plants for a couple of weeks, the adult may only feed on nectar for a few days, while moderately pollinating flowers. Ant species, instead, have very flexible feeding behaviors, which largely depend on the available feed sources around a nest. They can be purely carnivorous or omnivorous, even indirectly herbivorous when attending aphids. Although the present study may contain this bias, the most abundant species used for our calculations are comparatively well known, and their feeding behavior is well characterized.

The methodological decision to reduce our overall sample set to 24 species was critical, demands a careful trade-off, and justification, but allowed for a reliable ordination (NMDS, RDA). The analysis of only a fraction of the sampled species are, however, typical for ecological studies dealing with multivariate data analyses as it reduces noise and allows the drawing of more meaningful relationships [33,34,35,36].

In our study, we applied a quite rigorous reduction because we found only a few species that were heterogeneously distributed across the sampling locations but were still abundant. To our knowledge, there are only rules of thumb on how many species should be considered for an NMDS ordination and how their results are interpreted: (i) species used should not exceed the number of plots sampled, which was true for our case (24 species vs. 36 fields); (ii) abundances should be more or less equally distributed across groups, which was achieved by choosing only taxa that were present in at least three of the four irrigation water sources; (iii) rare species should be excluded, which was done by applying the ≥ 0.1% frequency criteria; lastly (iv) only stress values below 0.2 are considered acceptable for NMDS outputs. However, we also tested the most dominant 9 (all those with a frequency >1.5%), 15 (>1%), 20 (>0.5%), 29 (>0.4%), and found stress values of 0.15, 0.11, 0.12, and 0.17, respectively, where certain distortions were observed (except for *n =* 9), although the overall tendency of similarly behaving well- and wastewater (strong clustering and almost no overlap) locations were both confirmed.

The RDA analysis showed that our explanatory parameters—C/N, EC, P, and pH—explained only a very small part of the variation of the arthropod species data, and this was not statistically significant. Borcard et al. [78] recommended that such a result should not be interpreted nor plotted at all. However, since *p*-values are no measures of evidence, we showed the plots to allow the display of trends in otherwise very noisy data. A plausible explanation of low explanatory power could be that presence of arthropods is rather induced by volatile chemical components of the irrigation water than by the soil parameters we measured.

In general, our data indicated local effects of the irrigation water source, but we might have missed finding any relationship of the cultivated vegetable species on arthropod richness, diversity, and feeding guilds. Instead, several factors might have led to these rather heterogeneous outcomes:

First, we studied a highly mosaic-like, small-scale vegetable farming system characterized by a high spatial concentration of multiple crops with dozens of niches. This favors a homogenous distribution of most species in space and time, although changes in the dominance of specific feeding guild with crop type can be expected even in small-scale systems [79]. Plant species and their density affect arthropod communities significantly due to their variable plant architectures, specific traits, alternate microclimatic conditions, pest attractiveness, and nectar provision potential [80].

Second, most of the species observed are highly mobile and live under continuously changing agro-ecosystem conditions, such as planting and harvesting activities, different farmers’ practices, comparatively rapid crop cycles (lettuce), different life stages (all crops), variable fertilizer applications, and varying irrigation sources. All these lead to high turnovers and highly flexible habitat dwelling times. Hence, the timing and the extent of the sampling approach, including the length of monitoring and the choice of the season, have major effects on the catch and, therefore, the data. Jung et al. [81], for instance, suggested >28 days for sampling and assessing arthropod communities. Although we covered this period (August), the actual sample time per field was limited to three days. The choice of the season, on the other hand, might have affected feeding guilds like the pollinators, who are particularly abundant when crops are in bloom [82]. In our study, the main flowering period had happened already, which likely resulted in a comparatively low number of caught pollinator species. However, considering the comparatively high number of 36 fields (and more trap repetitions within), the chosen sampling intensity seems still reliable and technically feasible.

Third, also the sizes of the sampled vegetable fields and the sample area per location largely differed. The tap irrigated location *Gumbehene New Dam* was the largest sampling area, providing certainly also a wide range of niches. This effect appeared evident when considering the second largest count of species richness as well as the most spread NMDS coordinates for *Gumbehene New Dam*.

Lastly, our experimental setup did not allow for random distribution of the test fields and the water source that farmers used to irrigate their fields. Thus, our data was affected by spatial autocorrelation, preventing us from elucidating the relationship between wastewater irrigation and the prevalence of disease-transmitting insects, which may pose a risk to farmers’ health.

## 5. Conclusions

To our knowledge, this study is the first attempt to characterize the composition and functional partitioning of arthropod species communities sampled in West African UPA systems, in which farmers’ management strategies vary widely regarding irrigation water quality. Despite the sampling’s short duration, our results show an effect of wastewater use for irrigation on arthropod species and abundance with particular focus on the presence of critical species (invaders and disease vectors). To predict how the aforementioned and highly dynamic species communities will affect vegetable production and human livelihoods in urban areas of the Northern Region of Ghana, a heavily understudied area so far, biodiversity-monitoring schemes must be implemented at regional scales. These will allow to deliver long-term data and to monitor biodiversity change associated with land use, population pressure, and agricultural intensification.

## Figures and Tables

**Table 1:** Mean ± standard deviation of soil carbon-to-nitrogen ratio (C/N), electrical conductivity (EC), total phosphorous (P), pH, and counts of *Escherichia coli* and *Enterococcus* spp. (in most probable number (MPN) 100 mL^−1^) within the irrigation water source measured in vegetable fields under different irrigation sources in four urban and peri-urban agriculture (UPA) locations in Tamale, Ghana, in August 2016. Different letters indicate significant differences between groups at *p* ≤ 0.05. Note that fecal bacteria loads were only sampled once per location; hence, no standard deviations were calculated. WW = wastewater.

Irrigation	EC (µS cm^−1^)	C/N	P (g kg^−1^)	pH	*E. coli* (MPN 100 mL^−1^)	*Enterococcus* spp. (MPN 100 mL^−1^)
Rainfed	68.84 ± 33.75 ^a^	10.14 ± 1.50 ^a^	75.38 ± 77.98 ^a^	6.37 ± 0.70 ^ab^	-	-
Tap	129.01 ± 135.69 ^ab^	11.94 ± 2.80 ^ab^	73.25 ± 43.69 ^a^	5.92 ± 0.81 ^a^	4.23	3.71
Well	225.95 ± 98.52 ^ab^	12.73 ± 2.97 ^ab^	210.82 ± 101.90 ^ab^	6.67 ± 0.13 ^ab^	3.63	3.04
WW	280.83 ± 187.20 ^b^	13.71 ± 2.75 ^b^	94.64 ± 36.90 ^b^	6.70 ± 0.99 ^b^	6.57	5.83

**Table 2:** Total abundance, total species richness, and diversity indices based on Hill numbers of tree communities found in four UPA locations under different irrigation sources, namely, rainfed (RF), tap water (Tap), well water (Well), and wastewater (WW) in Tamale, Ghana, in August 2016. Note that tree species abundance and richness were only sampled once per location; hence, no standard deviations were calculated.

Location	Abundance	Species Richness	Exponential Shannon Index	Inverse Simpson’s Index	Berger–Parker Index
**Rainfed**	101	15	5.6	3.3	1.9
**Tap**	125	11	5.2	4.1	2.7
**Well**	78	13	6.9	5.7	3.3
**WW**	73	9	5.8	5.2	3.2

**Table 3:** Total abundance, total species richness, and diversity indices based on Hill numbers of arthropod communities found on vegetable fields in four UPA locations and with different irrigation water sources, namely, rainfed (RF), tap water (Tap), well water (Well), and wastewater (WW), in Tamale, Ghana in August 2016.

Location	Total Abundance	Species Richness	Exponential Shannon Index	Inverse Simpson’s Index	Inverse Berger–Parker Index
**Rainfed**	2105	90	19	9.36	3.89
**Tap**	3888	113	12	5.65	2.16
**Well**	2131	85	18	6.11	2.38
**WW**	6102	136	11	4.11	2.00

**Table 4:** Mean functional richness, evenness, and dispersion of arthropod communities as well as community-weighted mean (±standard deviation) of the five arthropod guilds recorded during a 72-h sampling, using the pan and pitfall traps in different vegetable fields in four UPA locations and with different irrigation water sources, namely, rainfed (RF), tap water (Tap), well water (Well), and wastewater (WW) in Tamale, Ghana in August 2016.

	Functional Richness	Functional Evenness	Functional Dispersion	CWM Carnivore	CWM Decomposers	CWM Herbivore	CWM Omnivore	CWM Pollinators
**Locations**								
**Rainfed**	4.71 ± 0.50	0.13 ± 0.02	0.27 ± 0.09	0.04 ± 0.03	0.11 ± 0.14	0.25 ± 0.18	0.58 ± 0.21	0.01 ± 0.01
**Tap**	3.90 ± 1.04	0.13± 0.06	0.23 ± 0.08	0.14 ± 0.21	0.24 ± 0.36	0.19 ± 0.24	0.41 ± 0.31	0.01 ± 0.02
**Well**	4.43 ± 0.53	0.14 ± 0.06	0.34 ± 0.05	0.13 ± 0.12	0.42 ± 0.20	0.26 ± 0.12	0.17 ± 0.17	0.01 ± 0.01
**WW**	4.55 ± 0.69	0.14 ± 0.06	0.31 ± 0.05	0.20 ± 0.22	0.40 ± 0.21	0.23 ± 0.19	0.16 ± 0.19	0.01 ± 0.01

**Table 5:** Ecological relevance and abundance of the 24 most abundant and equally distributed arthropod species found on vegetable fields under different irrigation sources, namely, rainfed (RF), tap water (Tap), well water (Well), and wastewater (WW) in Tamale, Ghana, in 2016.

Order	Family	Genus	Species	Distribution/Origin	Relevance	Rainfed	Tap	Well	WW	Total Abundance
Araneae	Agelenidae	*Agelenopsis*	*aperta*	Dry regions	Carnivore/Pest regulator	12	24	29	442	507
Coleoptera	Chrysomelidae	*Ootheca*	*mutabilis*	Afrotropic	Herbivore	10	17	4	66	97
		*Podagrica*	*sjostedti*	n.a.	Herbivore	15	8	90	50	163
	Scarabaeidae	*Sarcophaga*	*carnaria*	Worldwide	Decomposer	0	32	1	7	40
Diptera	Calliphoridae	*Calliphora*	sp.	n.a.	Decomposer	1	0	5	1	7
	Chironomidae	*Chironomus*	*plumosus*	Northern hemisphere	Decomposer	0	5	56	33	94
	Chloropidae	*Hippelates*	*pusio*	Nearctic, Neotropic	Decomposer/Vector	191	1120	817	2883	5011
	Muscidae	*Musca*	*domestica*	Worldwide	Decomposer	11	15	0	22	48
	Stratiomyidae	*Hermetia*	*illucens*	Worldwide	Decomposer	1	50	0	1	52
Hemiptera	Cicadellidae	*Empoasca*	*facialis*	Neotropic	Herbivore	36	41	60	105	242
	Delphacidae	*Javesella*	*pellucida*	Worldwide	Herbivore/Vector	29	0	10	14	53
	Miridae	*Lygus*	sp.	n.a.	Herbivore	0	6	9	3	18
	Pentatomidae	*Aspavia*	*armigera*	Neotropic	Herbivore	0	1	5	2	8
Hymenoptera	Apidae	*Apis*	*mellifera*	Worldwide	Herbivore	10	6	9	6	31
	Formicidae	*Camponotus*	sp.	n.a.	Omnivore	22	237	0	60	319
		*Monomorium*	*pharaonis*	Neotropic, Palaearctic	Omnivore	320	72	0	205	597
		*Solenopsis*	*xyloni*	USA	Omnivore	501	256	60	146	963
		*Tetramorium*	*caespitum*	Europe	Omnivore	169	0	121	392	682
	Megachilidae	*Megachile*	*latimanus*	Worldwide	Pollinator	0	5	2	1	8
	Scelionidae	*Eumicrosoma*	*beneficum*	Worldwide	Carnivore/Pest regulator	2	4	0	5	11
Lepidoptera	Crambidae	*Hellula*	*undalis*	Worldwide	Herbivore	15	17	16	8	56
	Hesperiidae	*Zophopetes*	*dysmephila*	Afrotropic	Herbivore	8	2	0	3	13
Orthoptera	Gryllidae	*Gryllus*	sp.	n.a.	Herbivore	5	4	27	44	80
	Tetrigidae	*Tetrix*	*subulata*	Worldwide	Herbivore	0	4	3	13	20

## Supplementary Materials

The following are available online at https://www.mdpi.com/2075-4450/11/8/488/s1, Table S1: List of tree species found in urban- and peri-urban vegetable fields under different irrigation sources in Tamale, Northern Region, Ghana in August 2016, Table S2: List of arthropod species found in urban- and peri-urban vegetable fields under different irrigation sources in Tamale, Northern Region, Ghana in August 2016.
