# Supplementary material for: Arthropod Communities in Urban Agricultural Production Systems under Different Irrigation Sources in the Northern Region of Ghana
Source: Insects. 2020 Aug 1;11(8):488. doi: 10.3390/insects11080488 (PMC7469155; doi:10.3390/insects11080488)
Supplement: Supplementary file 1 [file insects-11-00488-s001.zip › Table S1.docx]

**Table S1.** List of tree species found in urban- and peri-urban vegetable fields under different irrigation sources in Tamale, Northern Region, Ghana in August 2016.

| **Tree species** | **Rainfed**  **(Youngi-duuni)** | **Tap water**  **(Gumbehene New Dam)** | **Well water (Sangaani)** | **Wastewater (Nyanshegu)** |
| --- | --- | --- | --- | --- |
| ***Anogeissus leiocarpus*** | **3** | **0** | **0** | **0** |
| ***Acacia versillatum*** | **0** | **2** | **0** | **0** |
| ***Adansonia digitata*** | **3** | **0** | **0** | **0** |
| ***Albizia lebbeck*** | **12** | **36** | **3** | **16** |
| ***Anarcardium occidentale*** | **1** | **0** | **0** | **0** |
| ***Annona muricata*** | **0** | **0** | **1** | **0** |
| ***Azadirachta indica*** | **53** | **46** | **15** | **11** |
| ***Ceiba petandra*** | **5** | **9** | **4** | **8** |
| ***Citrus sinensis*** | **2** | **0** | **1** | **0** |
| ***Cocos nucifera*** | **1** | **0** | **3** | **0** |
| ***Delonix regia*** | **0** | **1** | **1** | **0** |
| ***Diospyros mespiliformis*** | **2** | **0** | **0** | **0** |
| ***Elaeis guineensis*** | **0** | **0** | **5** | **0** |
| ***Faidherbia albida*** | **2** | **0** | **0** | **0** |
| ***Ficus benjamina*** | **4** | **0** | **0** | **0** |
| ***Ficus glumosa*** | **0** | **0** | **0** | **1** |
| ***Ficus gnaphalorcarpa*** | **0** | **0** | **0** | **2** |
| ***Ficus sp.*** | **0** | **0** | **13** | **0** |
| ***Khaya senegalensis*** | **7** | **2** | **0** | **0** |
| ***Lagenaria siceraria*** | **0** | **0** | **2** | **0** |
| ***Lannea acida*** | **2** | **0** | **0** | **0** |
| ***Mangifera indica*** | **3** | **13** | **24** | **23** |
| ***Milletia thonningia*** | **0** | **2** | **0** | **0** |
| ***Moringa oleifera*** | **0** | **10** | **0** | **3** |
| ***Parkia biglobosa*** | **0** | **2** | **0** | **0** |
| ***Polyalthia longifolia*** | **0** | **0** | **5** | **0** |
| ***Tectona grandis*** | **0** | **2** | **0** | **0** |
| ***Terminalia catappa*** | **0** | **0** | **1** | **4** |
| ***Vitellaria paradoxa*** | **1** | **0** | **0** | **5** |
