# Supplementary material for: Arthropod Communities in Urban Agricultural Production Systems under Different Irrigation Sources in the Northern Region of Ghana
Source: Insects. 2020 Aug 1;11(8):488. doi: 10.3390/insects11080488 (PMC7469155; doi:10.3390/insects11080488)
Supplement: Supplementary file 1 [file insects-11-00488-s001.zip › Table S2.docx]

**Table S2.** List of arthropod species found in urban- and peri-urban vegetable fields under different irrigation sources in Tamale, Northern Region, Ghana in August 2016.

| **Orde** | **Family** | **Genus** | **Species** | **Guild** | **Authority** | **Rainfed** | **Tap** | **Wastewater** | **Well** | **Grand Total** |
| --- | --- | --- | --- | --- | --- | --- | --- | --- | --- | --- |
| Araneae | Agelenidae | *Agelenopsis* | *aperta* | Carnivore | Gertsch, 1934 | 12 | 24 | 442 | 29 | 507 |
|  | Desidae | *Badumna* | *insignia* | Carnivore | L. Koch, 1872 |  |  | 11 |  | 11 |
|  |  |  | sp. | Carnivore | Thorell, 1890 |  | 41 |  |  | 41 |
|  | Sparassidae | *Isopedella* | sp. | Carnivore | Hirst, 1990 |  | 2 |  |  | 2 |
|  |  | *Palystes* | *superciliosus* | Carnivore | L. Koch, 1875 |  | 1 |  |  | 1 |
| Coleoptera | Anthicidae | *Anthelephila* | *pedestris* | Carnivore | Rossi, 1790 | 1 |  | 3 |  | 4 |
|  | Aphididae | *Aphis* | *gossypii* | Herbivore | Glover, 1877 | 2 |  |  |  | 2 |
|  | Buprestidae | *Agrilus* | *derasofasciatus* | Herbivore | Boisduval & Lacordaire, 1835 |  |  |  | 1 | 1 |
|  |  | *Sternocera* | *interrupta* | Herbivore | Olivier, 1790 | 1 |  |  |  | 1 |
|  |  | *Sternoceras* | sp. | Herbivore | Eschscholtz, 1829 |  |  |  | 1 | 1 |
|  | Carabidae | *Agonum* | sp. | Carnivore | Linne, 1758 |  | 6 |  |  | 6 |
|  |  |  | *viduum* | Carnivore | Panzer, 1796 |  |  | 15 |  | 15 |
|  |  | *Amara* | *plebeja* | Herbivore | Gyllenhal, 1810 |  | 2 |  |  | 2 |
|  |  | *Bembidion* | *articulatum* | Carnivore | Panzer, 1796 |  | 1 |  |  | 1 |
|  |  |  | sp. | Carnivore | Latreille, 1802 |  |  | 1 | 4 | 5 |
|  |  | *Brachypeplus* | *pilosellus* | Herbivore | Murray, 1864 |  |  |  | 16 | 16 |
|  |  | *Bradycellus* | *ruficollis* | Herbivore | Stephens, 1828 |  | 3 |  | 3 | 6 |
|  |  |  | sp. | Herbivore | Erichson, 1837 |  | 1 |  |  | 1 |
|  |  | *Calathus* | *rotundicollis* | Carnivore | Dejean, 1828 |  |  | 4 |  | 4 |
|  |  | *Catascopus* | sp. | Carnivore | Kirby, 1825 |  |  | 1 |  | 1 |
|  |  | *Cicindela* | *nigrior* | Carnivore | Linnaeus, 1758 |  |  | 13 |  | 13 |
|  |  | *Colliuris* | sp. | Carnivore | De Geer, 1774 | 1 |  |  |  | 1 |
|  |  | *Galerita* | sp. | Carnivore | Fabricius, 1801 | 7 |  |  |  | 7 |
|  |  | *Hybothecus* | *flohri* | Herbivore | Bates, 1882 |  |  | 5 |  | 5 |
|  |  | *Panagaeus* | *bipustulatus* | Herbivore | Fabricius, 1775 |  | 2 |  |  | 2 |
|  |  | *Tachyta* | sp. | Herbivore | Kirby, 1837 |  |  |  | 2 | 2 |
|  | Certonidae | *Diplognatha* | *gagates* | Herbivore | Forster, 1771 |  | 1 |  |  | 1 |
|  | Chrysomelidae | *Acanthoscelides* | *obtectus* | n.a. | Say, 1831 |  | 2 |  |  | 2 |
|  |  | *Altica* | *oleracea* | Herbivore | Scott, 1876 | 4 |  |  |  | 4 |
|  |  | *Calomela* | *eyrei* | Herbivore | Blackburn, 1890 |  | 1 |  |  | 1 |
|  |  | *Cryptocephalus* | *sericeus* | Herbivore | Franz, 1938 |  | 1 |  |  | 1 |
|  |  | *Lema* | *daturaphila* | Herbivore | Kogan and Goeden, 1970 |  | 2 |  |  | 2 |
|  |  |  | sp. | Herbivore | Fabricius, 1798 | 4 |  |  |  | 4 |
|  |  | *Ootheca* | *mutabilis* | Herbivore | Sahlberg, 1829 | 10 | 17 | 66 | 4 | 97 |
|  |  | *Phyllotreta* | *undulata* | Herbivore | Kutschera, 1860 | 1 |  |  |  | 1 |
|  |  | *Podagrica* | *sjostedti* | Herbivore | Fabricius, 1794 | 15 | 8 | 50 | 90 | 163 |
|  |  |  | *uniformis* | Herbivore | Jacoby |  |  | 122 |  | 122 |
|  | Ciidae | *Cis* | *fuscipes* | Herbivore | Fabricius, 1794 | 1 |  |  |  | 1 |
|  | Coccinellidae | *Aphidecta* | sp. | Carnivore | Linnaeus, 1758 | 1 |  |  |  | 1 |
|  |  | *Cheilomenes* | *lunata* | Carnivore | Fabricius, 1775 | 2 | 1 |  |  | 3 |
|  |  | *Chilocorus* | *circumdatus* | Carnivore | Gyllenhall in Schönherr, 1808 |  |  | 1 |  | 1 |
|  |  | *Scymnus* | sp. | Carnivore | Kugelann 1794 |  |  |  | 1 | 1 |
|  | Corylophidae | *Orthoperus* | *atomus* | Decomposer | Gyllenhal, 1808 |  | 2 |  |  | 2 |
|  | Curculionidae | *Archarias* | *salicivorus* | Herbivore | Paykull, 1792 |  |  | 1 |  | 1 |
|  |  | *Bryochaeta* | *pusillus* | Herbivore | Pascoe, 1871 |  |  |  | 4 | 4 |
|  |  | *Curculio* | *glandium* | Herbivore | Marsham, 1802 | 2 |  |  |  | 2 |
|  |  | *Neomycta* | sp. | Herbivore | Pascoe, 1877 |  |  |  | 2 | 2 |
|  |  | *Xyleborus* | *nitidipennis* | Herbivore | Roubal, 1937 |  |  |  | 7 | 7 |
|  | Cyrambicidae | *Exocentrus* | sp. | Decomposer | Dejean, 1835 |  |  | 1 |  | 1 |
|  | Dermastidae | *Trogoderma* | *glabrum* | Decomposer | Herbst, 1783 |  |  | 2 |  | 2 |
|  | Elateridae | *Adrastus* | *pallens* | Herbivore | Fabricius, 1792 | 1 |  |  |  | 1 |
|  |  | *Athous* | *niger* | Herbivore | Linnaeus, 1758 |  | 1 |  |  | 1 |
|  |  |  | *vittatus* | Herbivore | Fabricius, 1793 |  |  |  | 1 | 1 |
|  |  | *Conoderus* | *lividus* | Herbivore | De Geer, 1774 |  | 1 |  |  | 1 |
|  |  | *Dalopius* | *marginatus* | Omnivore | Linnaeus, 1758 |  | 3 |  |  | 3 |
|  | Geotrupidae | *Geotrupes* | sp. | Decomposer | Latreille, 1797 |  |  | 1 |  | 1 |
|  |  |  | *splendidus* | Decomposer | Fabricius, 1775 |  | 1 |  |  | 1 |
|  | Histeridae | *Asolenus* | *julietteae* | Carnivore | Gomy, 2014 | 3 |  |  |  | 3 |
|  |  | *Hister* | *quadrimaculatus* | Decomposer | Linnaeus, 1758 |  |  |  | 2 | 2 |
|  |  | *Margarinotus* | *niponicus* | Decomposer | Lewis, 1895 |  |  | 1 |  | 1 |
|  |  |  | sp. | Carnivore | Marseul, 1853 |  |  | 2 |  | 2 |
|  |  | *Platylomalus* | sp. | Carnivore | Cooman, 1948 |  |  | 1 |  | 1 |
|  | Lamproblattidae | *Dictyoptera* | sp. | Omnivore | Melichar, 1912 |  | 2 |  |  | 2 |
|  | Meliodae | *Mylabris* | *variabilis* | Pollinator | Pallas, 1782 | 4 |  | 3 |  | 7 |
|  | Nitidulidae | *Epuraea* | *aestiva* | Herbivore | Linnaeus, 1758 | 51 |  |  |  | 51 |
|  |  |  | *alternans* | n.a. | Grouvelle, 1912 |  | 58 |  | 17 | 75 |
|  |  |  | *luteola* | Herbivore | Erichson, 1843 |  |  | 147 | 65 | 212 |
|  | Rhagionidae | *Rhagio* | *aterrimus* | Carnivore | Fabricius, 1775 | 4 |  |  |  | 4 |
|  | Scarabaeidae | *Chiron* | sp. | Decomposer | Macleay, 1819 |  |  | 3 |  | 3 |
|  |  | *Cyclocephala* | *lurida* | Herbivore | Bland, 1863 |  | 1 |  |  | 1 |
|  |  | *Diplotaxis* | sp. | Herbivore | Kirby, 1837 |  |  | 1 |  | 1 |
|  |  | *Omaloplia* | sp. | Herbivore | Schönherr, 1817 |  |  |  | 2 | 2 |
|  |  | *Onthophagus* | *coenobita* | Decomposer | Herbst, 1783 |  |  |  |  | 0 |
|  |  | *Phyllophaga* | sp. | Herbivore | Harris, 1827 | 1 |  |  |  | 1 |
|  |  | *Rhizotrogus* | sp. | Herbivore | Lepeletier & Serville, 1825 |  |  | 2 |  | 2 |
|  |  | *Sarcophaga* | *carnaria* | Decomposer | Linnaeus, 1758 |  | 32 | 7 | 1 | 40 |
|  |  |  | sp. | Decomposer | Meigen, 1826 |  |  | 2 |  | 2 |
|  |  | *Serica* | sp. | Herbivore | Macleay, 1819 |  | 2 |  |  | 2 |
|  | Silvanidae | *Oryzaephilus* | *surinamensis* | Herbivore | Linnaeus, 1758 |  |  | 2 |  | 2 |
|  | Staphylinidae | *Batrisodes* | *venustus* | Carnivore | Reichenbach, 1816 |  | 5 |  |  | 5 |
|  |  | *Dacnochilus* | *compactus* | Herbivore | Casey, 1905 |  | 1 |  |  | 1 |
|  |  | *Gabrius* | *nigritulus* | Carnivore | Gravenhorst, 1802 |  |  |  | 5 | 5 |
|  |  | *Leptacinus* | *intermedius* | Carnivore | Donisthorpe, 1935 |  |  | 15 | 15 | 30 |
|  |  | *Othius* | *punctulatus* | Carnivore | Goeze, 1777 |  | 1 |  |  | 1 |
|  |  | *Paederus* | *riparius* | Carnivore | Fabricius, 1775 |  |  |  | 4 | 4 |
|  |  | *Scopaeus* | *sulcicollis* | Decomposer | Stephens, 1833) |  |  |  | 1 | 1 |
|  |  | *Xantholinus* | *bicolor* | n.a. | Sharp, 1876 |  |  |  | 2 | 2 |
|  |  |  | *decorus* | Carnivore | Erichson, 1839 |  |  | 1 |  | 1 |
|  |  |  | *elegans* | Carnivore | Olivier, 1795 |  |  | 3 |  | 3 |
|  |  |  | *tricolor* | Carnivore | Fabricius, 1787 |  | 1 |  |  | 1 |
|  | Tenebrionidae | *Gonocephalum* | *simplex* | Herbivore | Fabricius, 1801 |  |  | 5 |  | 5 |
|  |  | *Selinus* | sp. | Herbivore | Mulsant & Rey, 1853 |  |  |  | 1 | 1 |
|  |  | *Tenebrio* | *molitor* | Herbivore | Linnaeus, 1758 | 1 | 3 |  |  | 4 |
|  |  |  | *obscurus* | Herbivore | Fabricius, 1792 |  |  |  | 3 | 3 |
|  |  | *Tenebrio* | sp. | n.a. | Linnaeus, 1758 | 2 |  |  |  | 2 |
| Dermaptera | Forficulidae | *Forficula* | *auricularia* | Omnivore | Linnaeus, 1758 | 1 |  | 1 |  | 2 |
| Dictyoptera | Ectobiidae | *Blattella* | *germanica* | Omnivore | Linnaeus, 1767 |  | 1 | 10 |  | 11 |
|  |  | *Deropeltis* | sp. | Herbivore | Burmeister, 1838 |  |  | 1 |  | 1 |
|  |  | *Periplaneta* | *americana* | Omnivore | Linnaeus, 1758 |  |  | 4 |  | 4 |
| Diptera | Acroceridae | *Ogcodes* | *pallipes* | Carnivore | Latreille in Olivier, 1812 |  |  | 5 |  | 5 |
|  | Agromyzidae | *Agromyza* | *lambi* | Herbivore | Hendel, 1923 |  |  | 2 |  | 2 |
|  |  | *Ophiomyia* | *simplex* | Herbivore | Loew, 1869 | 3 |  |  |  | 3 |
|  | Asilidae | *Efferia* | *pogonias* | Carnivore | Walker, 1866 | 4 |  |  |  | 4 |
|  | Calliphoridae | *Calliphora* | sp. | Decomposer | Brauer & Bergenstamm, 1889 | 1 |  | 1 | 5 | 7 |
|  |  |  | *vomitoria* | Decomposer | Linnaeus, 1758 |  |  |  | 55 | 55 |
|  |  | *Lucilia* | *sericata* | Decomposer | Meigen, 1826 |  | 18 | 9 |  | 27 |
|  | Chironomidae | *Chironomus* | *plumosus* | Decomposer | Linnaeus, 1758 |  | 5 | 33 | 56 | 94 |
|  |  | *Chlorops* | *pumilionis* | Herbivore | Bjerkander, 1778 |  | 155 |  |  | 155 |
|  | Chloropidae | *Hippelates* | *pusio* | Decomposer | Loew | 191 | 1120 | 2883 | 817 | 5011 |
|  |  |  | sp. | Decomposer | Lioy, 1864 |  | 1112 |  |  | 1112 |
|  | Dolichopodidae | *Condylostylus* | sp. | Carnivore | Bigot, 1859 |  | 3 |  |  | 3 |
|  |  | *Sciapus* | sp. | Carnivore | Zeller, 1842 |  |  | 8 |  | 8 |
|  | Drosophilidae | *Drosophila* | *melanogaster* | Decomposer | Meigen, 1830 |  |  |  | 2 | 2 |
|  | Ephydridae | *Paralimna* | *nubifer* | Herbivore | Cresson, 1929 |  | 2 |  |  | 2 |
|  | Luxanidae | *Minettia* | *lupulina* | Herbivore | Linnaeus, 1758 |  |  | 5 |  | 5 |
|  | Muscidae | *Atherigona* | sp. | Herbivore | Rondani, 1856 |  |  | 1 | 1 | 2 |
|  |  | *Fannia* | *canicularis* | Decomposer | Linnaeus, 1761 |  | 8 |  |  | 8 |
|  |  | *Musca* | *domestica* | Omnivore | Linnaeus, 1758 | 11 | 15 | 22 |  | 48 |
|  |  | *Stomoxys* | *calcitrans* | Carnivore | Linnaeus, 1758 |  |  |  | 2 | 2 |
|  | Mycetophilidae | *Mycetophila* | sp. | Omnivore | Meigen, 1803 |  | 8 |  |  | 8 |
|  | Stratiomyidae | *Hermetia* | *illucens* | Decomposer | Linnaeus, 1758 | 1 | 50 | 1 |  | 52 |
|  | Syrphidae | *Episyrphus* | *balteatus* | Pollinator | De Geer, 1776 |  |  | 4 |  | 4 |
|  |  | *Syrphus* | *ribesii* | Omnivore | Linnaeus, 1758 |  |  |  | 2 | 2 |
|  | Tachinidae | *Phryxe* | *vulgaris* | Carnivore | Fallén, 1810 |  | 3 |  | 6 | 9 |
|  |  | *Spoggosia* | *claripennis* | Herbivore | Macquart | 7 |  |  |  | 7 |
|  |  | *Tachina* | *grossa* | Carnivore | Linnaeus, 1758 |  |  |  | 4 | 4 |
|  |  |  | sp. | Carnivore | Meigen, 1803 |  |  | 17 | 12 | 29 |
|  | Tephritidae | *Ceratitis* | *capitata* | Herbivore | Wiedemann, 1824 | 2 |  |  |  | 2 |
|  |  |  | *inscripta* | Herbivore | Wiedemann, 1824 |  |  |  | 2 | 2 |
|  |  | *Rhagoletis* | sp. | Herbivore | Loew, 1862 |  |  | 3 |  | 3 |
|  |  | *Rhynencina* | sp. | Herbivore | Johnson, 1922 |  | 2 |  |  | 2 |
|  |  | *Trypeta* | sp. | Herbivore | Meigen, 1803 | 1 |  |  |  | 1 |
| Glomerida | Glomeridae | *Glomeris* | *marginata* | Decomposer | Villers, 1789 |  |  | 3 | 22 | 25 |
| Hemiptera | Alydidae | *Alydus* | *eurinus* | Herbivore | Linnaeus, 1758 |  | 1 |  |  | 1 |
|  |  |  | sp. | Herbivore | Fabricius, 1803 | 12 |  | 9 |  | 21 |
|  |  | *Riptortus* | *serripes* | Herbivore | Fabricius, 1775 |  |  | 1 |  | 1 |
|  |  | *Stenocoris* | *americana* | Herbivore | Ahmad, 1965 |  |  | 1 | 2 | 3 |
|  | Anthocoridae | *Orius* | *tantillus* | Carnivore | Motschulsky |  |  | 59 |  | 59 |
|  | Aphididae | *Brevicoryne* | *brassicae* | Herbivore | Linnaeus, 1758 |  | 3 |  |  | 3 |
|  | Aphrophoridae | *Aphrophora* | *salicina* | Herbivore | Goeze, 1778 |  |  | 2 |  | 2 |
|  |  | *Poophilus* | sp. | Herbivore | Stål, 1866 |  | 15 | 13 |  | 28 |
|  |  | *Ptyelus* | *flavescens* | Herbivore | Fabricius, 1794 |  |  | 15 | 39 | 54 |
|  |  |  | *grossus* | Herbivore | Fabricius, 1781 |  |  | 12 |  | 12 |
|  |  | *Lepyronia* | *quadrangularis* | Herbivore | Say, 1825 |  | 26 |  | 15 | 41 |
|  | Aradidae | *Aradus* | sp. | Decomposer | Fabricius, 1803 |  | 2 |  |  | 2 |
|  | Cicadellidae | *Allygus* | *modestus* | Herbivore | Scott, 1876 | 7 |  |  |  | 7 |
|  |  | *Amrasca* | *biguttula* | Herbivore | Ishida, 1912 | 7 |  | 45 |  | 52 |
|  |  | *Cicadella* | *viridis* | Herbivore | Franz, 1938 |  | 3 |  |  | 3 |
|  |  | *Cicadula* | *quadrinotata* | Herbivore | Franz, 1938 | 5 |  |  |  | 5 |
|  |  | *Empoasca* | *facialis* | Herbivore | Jacobi 1912 | 36 | 41 | 105 | 60 | 242 |
|  |  | *Erythroneura* | sp. | Herbivore | Fitch, 1851 |  |  |  | 4 | 4 |
|  |  | *Streptanus* | *sordidus* | Herbivore | Zetterstedt, 1828 | 15 |  |  |  | 15 |
|  |  | *Xerophloea* | *viridis* | Herbivore | Fabricius, 1794 | 7 |  |  |  | 7 |
|  |  | *Zyginidia* | *scutellaris* | Herbivore | Herrich-Schaeffer, 1838 | 20 |  |  |  | 20 |
|  | Cixiidae | *Oliarus* | *humilis* | Herbivore | Say, 1830 |  |  |  | 2 | 2 |
|  | Coreidae | *Anoplocnemis* | *fuscus* | Herbivore | Westwood, 1842 |  |  | 1 |  | 1 |
|  |  | *Cletus* | sp. | Herbivore | Stål, 1860 | 2 |  | 7 |  | 9 |
|  |  |  | *trigonus* | Herbivore | Thunberg, 1783 |  |  | 2 |  | 2 |
|  |  | *Coreus* | *marginatus* | Herbivore | Linnaeus, 1758 |  | 2 |  |  | 2 |
|  | Cydnidae | *Pangaeus* | *bilineatus* | Herbivore | Say, 1825 |  | 2 |  |  | 2 |
|  | Delphacidae | *Javesella* | *pellucida* | Herbivore | Fabricius, 1794 | 29 |  | 14 | 10 | 53 |
|  |  | *Kamendaka* | sp. | Herbivore | Distant, 1906 |  |  | 17 |  | 17 |
|  |  | *Sogatella* | sp. | Herbivore | Fennah, 1956 | 3 |  |  |  | 3 |
|  |  | *Stobaera* | *tricarinata* | Herbivore | Say, 1825 |  |  | 17 |  | 17 |
|  | Elateridae | *Aspavia* | *armigera* | Herbivore | Fabricius, 1775 |  |  | 3 |  | 3 |
|  | Fulgoridae | *Fulgora* | sp. | Herbivore | Linnaeus, 1767 |  |  |  |  | 0 |
|  | Lygaeidae | *Lygaeus* | *rivularis* | Herbivore | Germar, 1837 |  | 9 | 6 |  | 15 |
|  |  |  | sp. | Herbivore | Fabricius, 1794 |  |  | 1 |  | 1 |
|  |  | *Pseudolasius* | sp. | Omnivore | Brailovsky, 1982 |  |  |  | 25 | 25 |
|  |  | *Pseudopamera* | *aurivilliana* | Herbivore | Distant, 1892 | 11 |  |  |  | 11 |
|  |  | *Spilostethus* | *hospes* | Herbivore | Fabricius & J.C., 1794 |  |  | 7 |  | 7 |
|  |  |  | *saxatilis* | Herbivore | Scopoli, 1763 |  | 1 |  |  | 1 |
|  | Miridae | *Boxia* | *khayae* | Herbivore | China, 1943 |  |  |  | 8 | 8 |
|  |  | *Capsus* | *ater* | Herbivore | Linnaeus, 1758 | 3 |  |  |  | 3 |
|  |  | *Chamus* | *boxi* | Herbivore | China |  |  |  | 20 | 20 |
|  |  |  | sp. | Herbivore | Distant, 1904 |  |  |  | 9 | 9 |
|  |  | *Liocoris* | *tripustulatus* | Herbivore | Fieber, 1858 |  |  | 8 |  | 8 |
|  |  | *Lygus* | *lineolaris* | Herbivore | Palisot de Beauvois, 1818 |  | 2 |  |  | 2 |
|  |  |  | *neavei* | Herbivore | Poppius, 1914 |  | 5 |  |  | 5 |
|  |  |  | sp. | Herbivore | Hahn, 1833 |  | 6 | 3 | 9 | 18 |
|  | Mordellidae | *Glipa* | *oculata* | Herbivore | Linnaeus, 1758 | 1 |  |  |  | 1 |
|  | Pentatomidae | *Aspavia* | *armigera* | Herbivore | Fabricius, 1775 |  | 1 | 2 | 5 | 8 |
|  |  | *Nezara* | *viridula* | Herbivore | Linnaeus, 1758 |  |  | 1 |  | 1 |
|  |  | *Oebalus* | *pugnax* | Herbivore | Fabricius, 1775 |  |  | 5 | 2 | 7 |
|  |  | *Pentatoma* | *rufipes* | Herbivore | Linnaeus, 1758 |  | 1 |  |  | 1 |
|  |  | *Podisus* | *brevispinus* | Carnivore | Phillips, 1982 | 5 |  |  |  | 5 |
|  | Pyrrhocoridae | *Dysdercus* | sp. | Herbivore | Meigen, 1830 |  | 10 |  |  | 10 |
|  |  |  | *superstitiosus* | Herbivore | Fabricius, 1775 |  | 10 | 1 |  | 11 |
|  |  |  | *suturellus* | Herbivore | Herrich-Schaeffer, 1842 |  | 1 |  |  | 1 |
|  | Reduviidae | *Empicoris* | *vagabundus* | Carnivore | Linnaeus, 1758 |  |  |  | 1 | 1 |
|  |  | *Ricania* | *cervina* | Herbivore | Melichar, 1898 |  | 1 |  |  | 1 |
|  |  | *Triatoma* | *rubida* | Carnivore | Uhler, 1894 |  |  | 4 |  | 4 |
|  | Scutelleridae | *Sphaerocoris* | *annulus* | Herbivore | Fabricius, 1775 |  |  | 1 |  | 1 |
|  | Tingidae | *Tingidae* | sp. | Herbivore | Laporte, 1807 |  |  | 1 |  | 1 |
| Hymenoptera | Sphecidae | *Chalybion* | sp. | Omnivore | Dahlbom, 1843 |  |  | 1 |  | 1 |
|  |  | *Liris* | *beatus* | Carnivore | Cameron, 1889 |  |  | 20 |  | 20 |
|  |  | *Podalonia* | *hirsuta* | Carnivore | Scopoli, 1763 |  | 2 |  |  | 2 |
|  |  | *Sceliphron* | *caementarium* | Carnivore | Drury, 1773 |  | 2 |  |  | 2 |
|  |  |  | *curvatum* | Carnivore | Westwood, 1833 | 1 |  |  |  | 1 |
|  | Andrenidae | *Andrena* | sp. | Pollinator | Fabricius, 1775 |  | 2 |  |  | 2 |
|  |  | *Perdita* | sp. | Pollinator | Smith, 1853 |  | 1 |  |  | 1 |
|  | Apidae | *Apis* | *mellifera* | Pollinator | Linnaeus, 1758 | 10 | 6 | 6 | 9 | 31 |
|  |  | *Melipona* | sp. | Pollinator | Illiger, 1806 | 3 |  |  |  | 3 |
|  |  | *Meliponula* | *ferruginea* | Pollinator | Cockerell, 1934 | 1 |  |  |  | 1 |
|  |  |  | sp. | Pollinator | Cockerell, 1934 | 2 |  | 6 |  | 8 |
|  |  | *Nomada* | *maculata* | n.a. | Cresson, 1863 |  |  |  | 1 | 1 |
|  |  | *Xylocopa* | *violacea* | Pollinator | Linnaeus, 1758 |  | 1 |  |  | 1 |
|  | Bethylidae | *Cephalonomia* | sp. | Carnivore | Wiedemann, 1824 |  |  | 3 |  | 3 |
|  | Cabronidae | *Tachytes* | *panzeri* | Carnivore | Dufour, 1841 | 1 |  |  |  | 1 |
|  | Chalcididae | *Brachymeria* | sp. | Carnivore | Westwood, 1829 | 1 |  |  |  | 1 |
|  | Chrysididae | *Trichrysis* | *cyanea* | Carnivore | Franz, 1938 |  | 1 |  |  | 1 |
|  | Colletidae | *Hylaeus* | *brevicornis* | Pollinator | Nylander, 1852 | 2 |  |  |  | 2 |
|  |  |  | *modestus* | Pollinator | Say, 1837 |  | 1 |  |  | 1 |
|  | Crabronidae | *Tachytes* | *panzeri* | Carnivore | Dufour, 1841 | 1 |  |  |  | 1 |
|  | Evaniidae | *Hyptia* | sp. | Carnivore | Illiger, 1807 |  |  | 2 |  | 2 |
|  | Formicidae | *Acanthomyrmex* | *volcanus* | Herbivore | Wheeler, W.M., 1937 |  | 7 |  |  | 7 |
|  |  | *Camponotus* | *atriceps* | Omnivore | Smith, 1858 |  | 75 |  |  | 75 |
|  |  |  | *auriventris* | Omnivore | Emery, 1889 |  | 16 |  |  | 16 |
|  |  |  | *chrysurus* | Omnivore | Gerstäcker, 1870 |  | 123 |  | 15 | 138 |
|  |  |  | *importunus* | Omnivore | Forel, 1911 | 143 |  | 57 |  | 200 |
|  |  |  | *ligniperda* | Omnivore | Forel, 1911 |  | 18 |  |  | 18 |
|  |  |  | *pennsylvanicus* | Omnivore | De Geer, 1773 | 8 |  |  |  | 8 |
|  |  |  | sp. | Omnivore | Mayr, 1861 | 22 | 237 | 60 |  | 319 |
|  |  | *Cataulacus* | *erinaceus* | Omnivore | Stitz, 1910 | 1 |  |  |  | 1 |
|  |  | *Dorylus* | sp. | Carnivore | Fabricius, 1793 |  | 7 |  | 17 | 24 |
|  |  | *Formica* | *rufa* | Omnivore | Linnaeus, 1761 |  | 44 |  |  | 44 |
|  |  | *Hypoponera* | *monticola* | Omnivore | Mann, 1921 |  |  | 8 |  | 8 |
|  |  | *Lasius* | *claviger* | Omnivore | Roger, 1862 |  | 7 |  |  | 7 |
|  |  |  | *fuliginosus* | Omnivore | Latreille, 1798 |  | 12 |  |  | 12 |
|  |  | *Linepithema* | *humile* | Omnivore | Fabricius, 1794 | 47 |  |  |  | 47 |
|  |  | *Manica* | *rubida* | Omnivore | Latreille, 1802 |  | 5 |  |  | 5 |
|  |  | *Monomorium* | *biocolor* | Omnivore | Emery, 1877 |  | 1 |  | 146 | 147 |
|  |  |  | *pharaonis* | Omnivore | Linnaeus, 1758 | 320 | 72 | 205 |  | 597 |
|  |  | *Odontomachus* | *brunneus* | Carnivore | Patton, 1894 |  | 3 | 60 |  | 63 |
|  |  |  | sp. | Carnivore | Latreille, 1804 |  |  | 3 |  | 3 |
|  |  | *Pachycondyla* | *commutata* | Carnivore | Roger, 1860 |  |  |  | 55 | 55 |
|  |  |  | *rufipes* | Carnivore | Jerdon, 1851 | 3 |  | 3 |  | 6 |
|  |  |  | sp. | Carnivore | Smith, F., 1858 |  |  | 44 |  | 44 |
|  |  | *Pheidole* | *megacephala* | Omnivore | Fabricius, 1793 |  |  |  | 36 | 36 |
|  |  | *Pogonomyrmex* | *barbatus* | Omnivore | Smith, 1858 |  | 10 |  |  | 10 |
|  |  |  | *occidentalis* | Omnivore | Cresson, 1865 | 15 |  |  |  | 15 |
|  |  |  | sp. | Omnivore | Mayr, 1868 |  |  | 2 |  | 2 |
|  |  | *Polyrhachis* | *decemdentata* | Carnivore | André, 1889 |  |  |  | 27 | 27 |
|  |  | *Solenopsis* | sp. | Omnivore | McCook, 1879 |  |  |  | 57 | 57 |
|  |  |  | *xyloni* | Omnivore | McCook, 1879 | 501 | 256 | 146 | 60 | 963 |
|  |  | *Sphinctomyrmex* | sp. | Carnivore | Mayr, 1866 |  |  | 2 |  | 2 |
|  |  | *Tetramorium* | *caespitum* | Omnivore | Linnaeus, 1758 | 169 |  | 392 | 121 | 682 |
|  |  |  | sp. | Omnivore | Mayr, 1855 | 18 |  | 42 |  | 60 |
|  | Halictidae | *Agapostemon* | *virescens* | Pollinator | Fabricius, 1775 |  | 2 |  |  | 2 |
|  |  | *Lasioglossum* | *sordidum* | Pollinator | Smith, 1853 |  |  | 3 |  | 3 |
|  |  |  | sp. | Pollinator | Curtis, 1833 | 1 |  |  |  | 1 |
|  | Lygaeidae | *Taphropeltus* | *contractus* | Herbivore | Herrich-Schaeffer & G.H.W., 1835 |  |  | 7 |  | 7 |
|  | Megachilidae | *Anthidium* | *manicatum* | Pollinator | Linnaeus, 1758 | 1 |  |  | 1 | 2 |
|  |  |  | *punctatum* | Pollinator | Latreille, 1809 |  |  | 1 |  | 1 |
|  |  | *Anthocoris* | *confusus* | Carnivore | Reuter, 1884 | 2 |  | 29 |  | 31 |
|  |  |  | sp. | Carnivore | Fallen, 1814 | 6 |  |  |  | 6 |
|  |  | *Megachile* | *latimanus* | Pollinator | Say, 1823 |  | 5 | 1 | 2 | 8 |
|  |  |  | sp. | Pollinator | Latreille, 1802 | 1 |  | 5 |  | 6 |
|  |  | *Osmia* | *lignaria* | Pollinator | Say, 1837 |  |  | 3 |  | 3 |
|  | Pompilidae | *Anoplius* | *americanus* | Carnivore | Beauvois, 1811 |  | 14 |  |  | 14 |
|  |  | *Entypus* | sp. | Carnivore | Dahlbom, 1843 |  |  | 1 |  | 1 |
|  |  |  | *unifasciatus* | Carnivore | Say, 1828 |  |  | 9 |  | 9 |
|  | Scelionidae | *Aradophagus* | *fasciatus* | Carnivore | Ashmead, 1893 |  |  | 2 |  | 2 |
|  |  | *Calliscelio* | *gracilis* | Carnivore | Nixon, 1931 |  | 7 |  |  | 7 |
|  |  | *Eumicrosoma* | *beneficum* | Carnivore | Gahan, 1913 | 2 | 4 | 5 |  | 11 |
|  |  | *Scelio* | sp. | Carnivore | Latreille, 1805 | 2 |  |  |  | 2 |
|  | Sphecidae | *Sphex* | *maximiliani* | Carnivore | Kohl, 1890 |  | 3 |  |  | 3 |
|  |  |  | sp. | Carnivore | Christ, 1791 |  |  | 1 |  | 1 |
|  | Trichogrammatidae | *Megaphragma* | sp. | Carnivore | Timberlake, 1924 | 1 |  |  |  | 1 |
|  | Vespidae | *Polistes* | *exclamans* | Carnivore | Viereck, 1906 | 2 |  |  |  | 2 |
|  |  |  | *instabilis* | Carnivore | Saussure, 1853 | 1 |  |  |  | 1 |
|  |  |  | *parametricus* | Carnivore | Matthias Buck et al., 2012 |  |  | 1 |  | 1 |
|  |  |  | *rubiginosus* | Herbivore | Lepeletier, 1836 |  |  | 3 |  | 3 |
|  |  |  | sp. | Carnivore | Latreille, 1802 |  |  | 1 |  | 1 |
|  |  | *Ropalidia* | *cincta* | Herbivore | Lepeletier, 1836 |  |  |  | 1 | 1 |
|  |  | *Vespa* | *crabro* | Carnivore | Linnaeus, 1758 |  |  |  | 1 | 1 |
| Isopoda | Oniscidae | *Oniscus* | *asellus* | Decomposer | Linnaeus, 1758 |  |  |  |  | 0 |
| Lepidoptera | Arctiinae | *Arctiinae* | sp. | Herbivore | Leach, 1815 |  |  |  | 1 | 1 |
|  | Crambidae | *Crocidolomia* | *binotalis* | Herbivore | Zeller, 1852 |  |  | 4 |  | 4 |
|  |  | *Hellula* | *undalis* | Herbivore | Fabricius, 1794 | 15 | 17 | 8 | 16 | 56 |
|  |  | *Sylepta* | *derogata* | Herbivore | Fabricius, 1775 |  | 22 |  |  | 22 |
|  | Depressariidae | *Ptilobola* | *inornatella* | Herbivore | Walsingham, 1891 |  |  |  | 5 | 5 |
|  | Erebidae | *Melipotis* | *gubernata* | Herbivore | Walker, 1857 |  |  |  | 2 | 2 |
|  | Geometridae | *Scopula* | *calothysanis* | Herbivore | Herbulot, 1965 |  | 1 |  |  | 1 |
|  | Hesperiidae | *Fresna* | *nyassae* | Herbivore | Hewitson, 1878 |  |  | 2 |  | 2 |
|  |  | *Pyrgus* | *malvae* | Pollinator | Linnaeus, 1758 |  | 3 |  |  | 3 |
|  |  | *Zophopetes* | *dysmephila* | Herbivore | Trimen, 1868 | 8 | 2 | 3 |  | 13 |
|  | Noctuidae | *Dypterygia* | sp. | Herbivore | Stephens, 1829 |  |  | 1 |  | 1 |
|  |  | *Ericeia* | *sobria* | Herbivore | Walker, 1857 |  | 1 |  |  | 1 |
|  |  | *Prodenia* | *litura* | Herbivore | Fabricius, 1775 |  | 1 |  |  | 1 |
|  |  | *Sesamia* | *calamistis* | Herbivore | Guenée, 1852 |  |  | 1 |  | 1 |
|  | Nolidae | *Earias* | *biplaga* | Herbivore | Walker, 1866 | 1 |  |  |  | 1 |
|  | Nymphalidae | *Acraea* | *eponina* | Pollinator | Cramer, 1780 |  |  | 1 |  | 1 |
|  |  | *Amauris* | *psyttalae* | Herbivore | Strand, 1913 |  | 1 |  |  | 1 |
|  |  | *Aterica* | *galene* | Decomposer | Brown, 1776 |  |  |  | 1 | 1 |
|  |  | *Acraea* | *eponina* | Pollinator | Cramer, 1780 | 1 |  |  |  | 1 |
|  | Pieridae | *Appias* | *epaphia* | Pollinator | Cramer, 1779 |  |  | 1 |  | 1 |
|  |  | *Colotis* | sp. | Herbivore | Hübner, 1819 |  | 1 |  |  | 1 |
|  |  | *Nepheronia* | *thalassina* | Herbivore | Boisduval, 1836 | 1 |  | 1 |  | 2 |
|  | Sphingidae | *Daphnis* | *nerii* | Pollinator | Linnaeus, 1758 |  |  | 1 |  | 1 |
|  |  | *Sphinx* | *chersis* | Herbivore | Hübner, 1823 | 1 |  |  |  | 1 |
|  |  | *Tinostoma* | sp. | Herbivore | Rothschild & Jordan, 1903 | 1 |  |  |  | 1 |
| Orthoptera | Acrididae | *Acrida* | *conica* | Herbivore | Fabricius, 1781 | 1 |  |  |  | 1 |
|  |  | *Chorthippus* | *dorsatus* | Herbivore | Zetterstedt, 1821 |  |  | 1 |  | 1 |
|  |  | *Orchelimum* | *gladiator* | Carnivore | Gyllenhal, 1808 |  |  |  | 1 | 1 |
|  |  | *Pholidoptera* | sp. | Carnivore | Wesmael, 1838 |  |  |  | 7 | 7 |
|  |  | *Phymateus* | *viridipes* | Herbivore | Stål, 1873 |  | 1 |  |  | 1 |
|  |  | *Ruspolia* | *indica* | Herbivore | Redtenbacher, 1891 |  |  |  | 8 | 8 |
|  |  | *Schistocerca* | *nitens* | Herbivore | Thunberg, 1815 |  | 3 |  |  | 3 |
|  | Gryllidae | *Acheta* | *domesticus* | Herbivore | Linnaeus, 1758 | 2 |  |  |  | 2 |
|  |  | *Brachytrupes* | *membranaceus* | Herbivore | Drury, 1770 |  |  | 3 |  | 3 |
|  |  | *Gryllus* | *lucens* | Herbivore | Walker, 1869 |  |  | 9 | 3 | 12 |
|  |  |  | sp. | Herbivore | Linnaeus, 1758 | 5 | 4 | 44 | 27 | 80 |
|  |  | *Teleogryllus* | *marini* | Herbivore | Otte, D. & R.D. Alexander, 1983 |  |  | 1 |  | 1 |
|  | Tetrigidae | *Tetrix* | *arenosa* | Herbivore | Burmeister, 1838 |  |  | 5 | 2 | 7 |
|  |  |  | sp. | Herbivore | Latreille, 1802 |  | 2 | 11 |  | 13 |
|  |  |  | *subulata* | Herbivore | Linnaeus, 1761 |  | 4 | 13 | 3 | 20 |
|  | Tettigoniidae | *Neoconocephalus* | *ensiger* | Herbivore | Harris, 1841 |  |  | 6 |  | 6 |
|  |  |  | *triops* | Herbivore | Linnaeus, 1758 |  |  | 1 |  | 1 |
|  |  | *Scudderia* | *furcata* | Omnivore | Brunner von Wattenwyl, 1878 |  | 1 |  |  | 1 |
| Phasmatodea | Phasmatidae | *Phobaeticus* | sp. | Herbivore | Brunner von Wattenwyl, 1907 |  |  | 2 |  | 2 |
| Thysanoptera | Thripidae | *Frankliniella* | *occidentalis* | Herbivore | Pergande, 1895 |  |  | 35 |  | 35 |
|  |  |  | *schultzei* | Herbivore | Trybom, 1910 | 122 |  |  |  | 122 |
|  |  |  | sp. | Herbivore | Fitch, 1855 | 58 |  |  |  | 58 |
|  |  |  | *tritici* | Herbivore | Fitch, 1855 |  | 6 |  | 15 | 21 |
|  |  | *Megalurothrips* | *sjostedti* | Herbivore | Trybom, 1908 | 85 |  | 342 |  | 427 |
|  |  | *Selenothrips* | *rubrocinctus* | Herbivore | Giard, 1901 |  |  |  | 10 | 10 |
|  |  | *Thrips* | *tabaci* | Herbivore | Lindeman, 1889 |  |  |  | 1 | 1 |
|  | Trichogrammatidae | *Megalurothrips* | *sjostedti* | Herbivore | Trybom, 1908 |  |  | 162 |  | 162 |
